# The link between social comparison orientation and domain-specific risk-taking: exploring the mediating role of two dimensions of trait competitiveness

**DOI:** 10.3389/fpsyg.2024.1340009

**Published:** 2024-06-04

**Authors:** Yuqian Wang, Andrew J. Elliot, Edmund Derrington, Yansong Li

**Affiliations:** ^1^Reward, Competition and Social Neuroscience Lab, Department of Psychology, School of Social and Behavioral Sciences, Nanjing University, Nanjing, China; ^2^Department of Psychology, University of Rochester, Rochester, NY, United States; ^3^Institute of Cognitive Science Marc Jeannerod, CNRS, Lyon, France; ^4^Institute for Brain Sciences, Nanjing University, Nanjing, China; ^5^Department of Radiology, The Affiliated Drum Tower Hospital, Nanjing University Medical School, Nanjing, China

**Keywords:** social comparison orientation, trait competitiveness, HCO, SDCO, domain-specific, risk-taking

## Abstract

**Introduction:**

Our recent research has demonstrated that social comparison orientation of ability (SCO-ability) is an antecedent of trait competitiveness (TC), and TC mediates the relation between SCO-ability and domain-specific risk-taking. TC is a multi-dimensional trait, therefore we sought to expand on prior research by examining whether SCO-ability predicted two distinct dimensions of TC: hypercompetitive orientation (HCO) and self-development competitive orientation (SDCO).

**Methods:**

We investigated how these different dimensions of TC mediated the relation between SCO-ability and both overall and domain-specific risk-taking in two correlational studies of 622 college students (313 males, mean age = 22.10, SD = 2.35) and 717 adult workers (368 males, mean age = 27.92, SD = 5.11).

**Results:**

We found that SCO-ability positively predicted HCO. Together, SCO-ability and HCO predicted overall risk-taking and risk-taking in the recreational and ethical domains in both samples. HCO mediated the relation between SCO-ability and both overall risk-taking and risk-taking in the recreational and ethical domains. Additionally, SCO-ability positively predicted SDCO. SCO-ability and SDCO mainly predicted risk-taking in the recreational domain in both studies. SDCO mediated the relation between SCO-ability and risk-taking only in the recreational domain.

**Discussion:**

Collectively, the findings above advance our understanding of the relation between competition and risk-taking by using differentiated measures of TC (HCO and SDCO). Our findings suggest that HCO is more strongly related to risk-taking than SDCO, thereby refining the possible role of SCO-ability and TC in predicting overall risk-taking and domain-specific risk-taking.

## 1 Introduction

According to the classic social comparison theory (Festinger, 1954), individuals have a dispositional tendency to compare themselves to others. Gibbons and Buunk (1999) coined the term “social comparison orientation” (SCO) to refer to individual variation in the tendency toward comparison with others. Given that social comparison is a process by which people assess themselves and come to understand themselves, their tendency to engage in social comparison with others may appear to be related to their dispositional preference to compete with others. Trait competitiveness (TC) measures such individual differences in the extent to which people compete with others (Elliot et al., 2018). Because both TC and SCO focus on the difference between self and others (Gardner et al., 2002; Diel et al., 2021; Reese et al., 2022), understanding the potential relation between them has been a focus of experimental inquiry over the past decades (Garcia et al., 2013, 2020; Chen et al., 2021; Garcia and Tor, 2024). Drawing on the existing literature, Garcia et al. (2013) proffered a theoretical framework that suggests that individuals’ competitiveness should be considered as one manifestation of the social comparison process and that social comparison with others can facilitate individuals’ competitiveness. According to this framework, we expect that individual variations in the extent to which people compare themselves with others may act as a stable indicator of individuals’ trait competitiveness (TC).

Recent work provides direct empirical evidence in support of the argument above by exploring the interrelation between SCO and TC (Liu et al., 2021). It is commonly recognized that SCO has two distinct dimensions of social comparison: ability and opinion (Gibbons and Buunk, 1999). SCO-ability is competition-based and focuses on comparing individuals’ skills and performance as determined by relative rank, which highlights a dispositional drive to outperform others (Festinger, 1954; Gibbons and Buunk, 1999; Suls et al., 2002; Elliot et al., 2018). When individuals take part in this form of comparison, they perceive the comparison targets as competitors (Park and Baek, 2018) and evaluate whether their performance or achievements surpass or fall short of those of the targets (Yang et al., 2018). In contrast, SCO-opinion is noncompetitive and compares individuals’ opinions, values, and beliefs with a focus on consensus accuracy (Wood, 1996; Suls et al., 2000; Yang and Robinson, 2018). During the information-based social comparison of opinion, individuals regard the comparison targets as informants, consultants, or role models, using their insights as a source of guidance or inspiration (Park and Baek, 2018; Yang et al., 2018). Given the noticeable conceptual overlap, it appears reasonable to assume that SCO-ability and TC will be positively correlated. Despite the progress made in this field, several issues remain to be addressed regarding the relation between SCO-ability and TC. For instance, TC should not be viewed as a global form of construct, but rather a multi-dimensional construct that describes different aspects within the construct of competitiveness (Ryckman et al., 1990; Houston et al., 2002; Newby and Klein, 2014). Thus, two distinct dimensions of TC have consistently been identified: hypercompetitiveness and personal development competitive attitude (Ryckman et al., 1990, 1996; Orosz et al., 2018). Hypercompetitiveness refers to the attitude of individuals who have a very strong need to compete and to win at any cost. In contrast the primary focus of personal development competitive attitude concerns personal growth and the enjoyment and mastery of a task in a competitive situation (Ryckman et al., 2009; Orosz et al., 2018). Thus, the assessment of general competitiveness, without considering its sub-dimensions, fails to fully capture the essence of the relation between SCO-ability and TC. For this reason, it is necessary to refine the relation between SCO-ability and these two distinct dimensions of TC. Therefore, the first aim of this research was to examine the extent to which SCO-ability is linked to hypercompetitiveness and personal development competitive attitude.

Propensity for risk-taking is highly sensitive to social factors. Therefore, an interesting line of research is to examine SCO and TC as predictors of risk-taking. Risk-taking refers to one’s behavioral tendency to pursue immediate rewards while disregarding potential negative consequences (Özmen and Sümer, 2011; Smith et al., 2015). However, research to link SCO and risk-taking remains limited. An early study showed a positive correlation between SCO and reckless driving (Gibbons and Gerrard, 1995), whereas a more recent study found that SCO negatively predicted substance abuse (Piko et al., 2010). Such mixed results seem to indicate that individuals with high SCO may either worry about being negatively evaluated by others, and therefore choose more cautious decision-making options, or in direct contrast, may take risks to obtain the corresponding social rewards.

In contrast, although evidence is relatively limited, the existing research on TC and risk-taking shows relatively reproducible results. Risk-sensitivity theory suggests that when individuals perceive themselves as being at a competitive disadvantage, they are more likely to engage in riskier behaviors as a strategic means to improve their relative positions (Mishra et al., 2014). In the context of competition—where success is often measured not by absolute standards, but relative to others—individuals who feel unable to succeed through safe, low-risk strategies may resort to higher-risk options (Daly and Wilson, 2001). This is evident in social and economic arenas, individuals who find themselves at a disadvantage due to lower social status or less access to resources (e.g., those who are unemployed or perceive themselves as less attractive) may adopt riskier behaviors as a means to achieve success or improve their standing (Wilson and Daly, 1997; Raphael and Winter-Ebmer, 2001). These decisions are driven by the calculus that the potential gains from taking risks outweigh the status quo’s likely losses, which aligns with the principles of risk-sensitivity theory. Engaging in high-risk behavior in such scenarios can be seen as a rational choice under the circumstances, shaped by evolutionary pressures to maximize competitive advantage despite potential costs. Previous studies found that males with higher competitiveness tend to seek attention by showing a higher propensity for risk-taking (Wilson and Daly, 2004). Consistent with this idea, Chen et al. (2015) showed that individuals with high competitiveness increased their propensity for risk-taking in the presence of a person of the same gender, because they sensed that their potential interests may be threatened. This motivated them to choose more risky options to protect their interests. Related studies also identified a positive correlation between TC and individuals’ gambling (Chiu and Storm, 2010; Harris et al., 2015).

The existing literature on SCO and TC as predictors of risk-taking has focused almost exclusively on risk-taking in the health/safety and gambling domains. However, the realms of social investment, recreation, and ethics are additional domains in which risk-taking has previously been specifically studied (Weber et al., 2002; Hu and Xie, 2012). Since an individual’s propensity for risk-taking can vary across domains, research that attends to the utility of SCO and TC in predicting risk-taking across multiple domains is clearly needed. A recent study contributes to our understanding of this issue (Liu et al., 2021). The principal outcomes of the study revealed that both SCO-ability and TC were significant predictors of not only general propensity for risk-taking but also specific behaviors in the realms of ethical, gambling, and health/safety risks. Additionally, the mediation analysis demonstrated that TC served as an intermediary in the relationship between SCO-ability and the propensity for risk-taking across both overall risk-taking and in the specific areas of ethical, gambling, and health/safety risks. In contrast, SCO-opinion exhibited few consistent relations to risk-taking. Despite such promising findings, it is still unclear how SCO-ability, hypercompetitiveness and personal development competitive attitude are linked to both overall and domain-specific risk-taking. Thus, the second aim of our present research was to test SCO-ability and these two distinct dimensions of TC as predictors of overall and domain-specific risk-taking and how hypercompetitiveness/personal development competitive attitude would mediate the relation between SCO-ability and overall and domain-specific risk-taking.

Based on those considerations, the present study aimed to elucidate the link between SCO, TC, and risk-taking by (1) examining whether SCO-ability predicted two distinct dimensions of TC; (2) investigating the utility of SCO-ability and these two different measures of TC, in predicting both overall and domain-specific risk-taking, and exploring the mediating role of two differentiated measures of TC in the relation between SCO-ability and risk-taking in two correlational studies in separate samples of college students or adult workers. Based on the current body of literature (Liu et al., 2021), we hypothesize that SCO-ability positively correlates with HCO/SDCO. We also anticipate that HCO is more strongly related to risk-taking than SDCO, as argued in previous research (Harris et al., 2015; Liu et al., 2021). Furthermore, as demonstrated in our recent study (Liu et al., 2021), we expect that HCO plays a mediating role in the effect of SCO-ability on domain-general and domain-specific risk-taking. In contrast, given that risk-taking in the recreational domain is driven by intrinsic motivation, and consequently has been shown to be chosen for promoting personal growth and well-being (Gracia-Garrido et al., 2022; Hernández-Méndez et al., 2024), we, in turn, hypothesize that SDCO mainly plays a mediating role in the link between SCO-ability and risk-taking behaviors in the recreational domain.

## 2 Study 1

### 2.1 Method

#### 2.1.1 Participants and procedure

In this study, we recruited a total of 622 university students aged between 18 and 25 years (313 males, with a mean age of 22.10 and a standard deviation of 2.35), through Wenjuanxing (WJX), an extensively used online questionnaire survey system in China. The target sample size was predetermined, following the procedure used in a prior study (Liu et al., 2021), adhering to the guideline suggested by Thompson (2000), which recommends a minimum ratio of variables to participants between 1:10 and 1:15. In this and subsequent studies, our sample size exceeded the number estimated according to this guideline, ensuring the robustness of our research. Data quality control was implemented through both system and manual checks. The WJX system filtered out respondents with duplicate IP addresses, devices, or incorrect answers to control questions (e.g., “What is the capital of China?”). Manual screening by WJX staff eliminated participants with abnormal completion times (either too long or too short-e.g., exclude reaction times greater than two standard deviation), inconsistent answers (e.g., contradictory responses to similar items), or signs of inattention (e.g., identical responses across a measure). Consequently, the aforementioned participant count represents the final sample size post data quality assurance. In these studies, no manipulations were made, all variables analyzed were disclosed, and all data collection and exclusions were completed prior to the analysis. Participants received 16 RMB (approximately $2) for completing an online survey in the specified order. Written informed consent was obtained from all participants before participation. The study received approval from the Ethical Review Board of Nanjing University.

#### 2.1.2 Measures (see Tables 1, 2 for descriptive statistics and Cronbach’s alphas)

##### 2.1.2.1 SCO

The Chinese version of the SCO scale was employed to assess individuals’ propensity to engage in social comparison (Wang et al., 2006). It comprised 11 items, rated on a 5-point Likert scale ranging from 1 (Strongly disagree) to 5 (Strongly agree). This scale assessed the following two dimensions of SCO: SCO-ability (e.g., “If I want to find out how well I have done something, I compare what I have done with how others have done”) and SCO-opinion (e.g., “I often try to find out what others believe, who face similar problems as what I face”).

##### 2.1.2.2 Hypercompetitiveness and personal development competitive attitude

The Chinese version of the Multidimensional Competitive Orientation Inventory (MCOI) was used to measure individual differences in hypercompetitiveness, namely hypercompetitive orientation (HCO) (e.g., “The most important thing is winning, no matter what”) and personal development competitive attitude, namely self-development competitive orientation (SDCO) (e.g., “I enjoy competition because it allows me to discover my abilities”) (Wang et al., 2024). The Chinese version demonstrates comparable validity and reliability with the original English version of the MCOI (Orosz et al., 2018). It included 12 items, rated on a 6-point Likert scale ranging from 1 (Not true of me at all) to 6 (Completely true of me). It is a multidimensional scale and thus the scores of these two dimensions cannot be combined.

##### 2.1.2.3 Risk-taking

The assessment of propensity for risk-taking in our research was conducted using the Chinese version of the domain-specific risk-taking scale (Hu and Xie, 2012). This instrument includes 35 questions, each answered on a 5-point Likert scale, where 1 signifies “very unlikely” and 5 “very likely.” The scale evaluates risky actions across five distinct areas: social-investment (for example, “Debating with a friend who holds a significantly different viewpoint”), recreational (such as “Venturing into an unfamiliar part of a city”), ethical (like “Imitating someone else’s signature”), gambling (“Wagering a week’s earnings in a casino”), and health/safety (“Riding a motorbike without a helmet”). A composite score for risk-taking was calculated by aggregating the scores from these areas.

### 2.2 Data analytic strategies

Initially, we conducted the descriptive statistics and bivariate correlations for the study variables with SPSS 26.0. Then we used path models to test the relation between two types of social comparison orientation (SCO-ability and SCO-opinion), two differentiated measures of TC (HCO and SDCO), and both overall and domain-specific risk-taking in Mplus 8.0^1^. As explained by recent research (Liu et al., 2021), a relation between SCO-opinion and TC was not expected, so the link between SCO-opinion and HCO was not examined in the present study. Concerning directionality in the interrelations between SCO-ability and TC, SCO-ability has been demonstrated to be an antecedent of TC (Liu et al., 2021). For this reason, the directional relation from SCO-ability to HCO/SDCO was tested in the present study. For either HCO or SDCO, there are 6 models that were tested using path analysis with observed variables, which corresponds to overall risk-taking and risk-taking in five domains. We used gender as a control variable on both the competition variable and risk-taking variable in all models. Taking into account that bootstrapping offers numerous benefits compared to traditional methods in analyzing mediation models (Preacher and Hayes, 2008), we employed the bootstrapping method (*N* = 10,000) with 95% confidence intervals (CI) to evaluate both the indirect and direct impacts of HCO or SDCO on the relationship between SCO-ability and overall, as well as domain-specific, propensity for risk-taking.

### 2.3 Results

#### 2.3.1 Relation between SCO, HCO, and risk-taking

**Figure 1:**
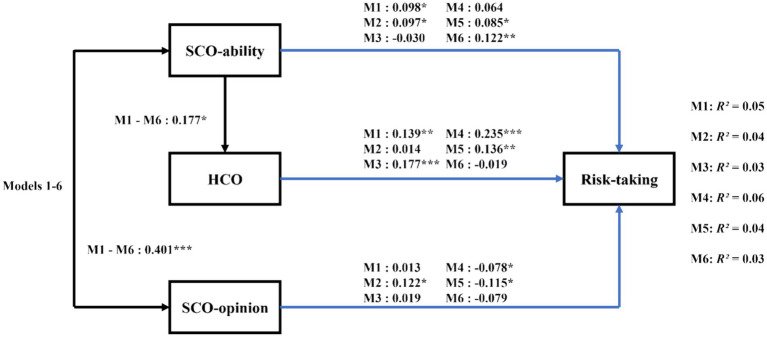
Diagram of Study 1: standardized path analysis for hypercompetitive orientation. Black pathways illustrate findings consistent across all risk-taking categories; blue pathways depict findings specific to each risk category: M1 for total risks, M2 for Social investment risks, M3 for recreational risks, M4 for ethical risks, M5 for gambling risks, M6 for health risks. R^2^ indicates the variance proportion in each risk type explained by the independent variables in each respective model. Sex was included in the study but not displayed in this figure for clarity. Solid lines represent significant paths (*p < 0.05, **p < 0.01, ***p < 0.001). SCO, social comparison orientation; HCO, hypercompetitive orientation.

Table 1 displays the bivariate correlations between the studied variables. Our analysis involved path analysis with observed variables across six proposed models. Each model demonstrated adequate compatibility with the data, and there was a high degree of similarity in fit among these models (e.g., overall risk-taking: χ^2^(2) = 4.20, *p =* 0.02, CFI = 0.97, SRMR = 0.02, RMSEA = 0.07). Overall, across all models, there was a strong correlation between SCO-ability and SCO-opinion. SCO-ability was a significant predictor of the HCO variable, and collectively, SCO-ability, SCO-opinion, and HCO were predictors of the risk-taking variable (Figure 1). Specifically, the results revealed that SCO-ability positively predicts HCO (*B* = 0.177, *SE* = 0.083, *t* = 2.125, *p* = 0.034, 95%CI = [0.015, 0.337]). SCO-ability positively predicted overall risk-taking (*B* = 0.098, *SE* = 0.044, *t* = 2.225, *p* = 0.026, 95%CI = [0.009, 0.122]), as well as risk-taking in the social investment (*B* = 0.097, *SE* = 0.047, *t* = 2.069, *p* = 0.039, 95%CI = [0.008, 0.192]), gambling (*B* = 0.085, *SE* = 0.040, *t* = 2.107, *p* = 0.035, 95%CI = [0.005, 0.164]) and health/safety (*B* = 0.122, *SE* = 0.046, *t* = 2.658, *p* = 0.008, 95%CI = [0.030, 0.209]) domains (Figure 1). SCO-opinion positively predicted risk-taking in the social investment domain (*B* = 0.122, *SE* = 0.047, *t* = 2.580, *p* = 0.010, 95%CI = [0.032, 0.218]), but negatively predicted risk-taking in the gambling (*B* = −0.115, *SE* = 0.045, *t* = −2.567, *p* = 0.010, 95%CI = [−0.203, −0.028]) and ethical domains (*B* = −0.078, *SE* = 0.039, *t* = −1.997, *p* = 0.046, 95%CI = [−0.157, −0.004]). HCO positively predicted overall risk-taking (*B* = 0.139, *SE* = 0.045, *t* = 3.067, *p* = 0.002, 95%CI = [0.048, 0.226]), as well as risk-taking in the recreational (*B* = 0.177, *SE* = 0.044, *t* = 4.033, *p* = 0.000, 95%CI = [0.089, 0.260]), ethical (*B* = 0.235, *SE* = 0.052, *t* = 4.535, *p* = 0.000, 95%CI = [0.132, 0.335]), and gambling (*B* = 0.136, *SE* = 0.045, *t* = 3.045, *p* = 0.002, 95%CI = [0.050, 0.224]) domains (Figure 1).

**Table 1:** Study 1: means, standard deviations, Cronbach’s alpha coefficients, and the bivariate relations among the variables from the hypercompetitive orientation model on student sample (*N* = 622).

	1	2	3	4	5	6	7	8	9
SCOA									
SCOO	0.396***								
HCO	0.276***	0.042							
(R_all)	0.139**	0.062	0.185***						
(R_socialinvestment)	0.147**	0.166***	0.064	0.771***					
(R_recreational)	0.026	0.016	0.173***	0.751***	0.412***				
(R_ethical)	0.099*	−0.046	0.238***	0.599***	0.197***	0.347***			
(R_gambling)	0.076	−0.072	0.166***	0.405***	−0.019	0.304***	0.384***		
(R_health)	0.083*	−0.022	0.045	0.619***	0.331***	0.298***	0.387***	0.178***	
Mean (*SD*)	3.37(0.72)	3.88(0.67)	2.65(1.04)	2.56(0.49)	3.32(0.73)	2.34(0.72)	1.75(0.63)	1.78(0.80)	2.70(0.78)
Cronbach’s alpha	0.74	0.64	0.78	0.86	0.85	0.70	0.71	0.75	0.55

SCOA, Social comparison orientation – ability; SCOO, Social comparison orientation – opinion; HCO, hypercompetitive orientation; R, Risk-taking; *p < 0.05, **p < 0.01, ***p < 0.001.

Additionally, our advanced bias-corrected bootstrap method, applied to evaluate the indirect effects of SCO-ability on various propensity for risk-taking via HCO, revealed significant indirect impacts. Specifically, the effect of SCO-ability on overall risk-taking was found to be significant (*B* = 0.025, *SE* = 0.015, 95%CI = [0.004, 0.063]), as well as its effects on recreational risk-taking (*B* = 0.031, *SE* = 0.017, 95%CI = [0.005, 0.073]), ethical risk-taking (*B* = 0.041, *SE* = 0.022, 95%CI = [0.006, 0.096]), and gambling risk-taking (*B* = 0.024, *SE* = 0.014, 95%CI = [0.004, 0.063]) through HCO.

#### 2.3.2 Relation between SCO, SDCO, and risk-taking

**Figure 2:**
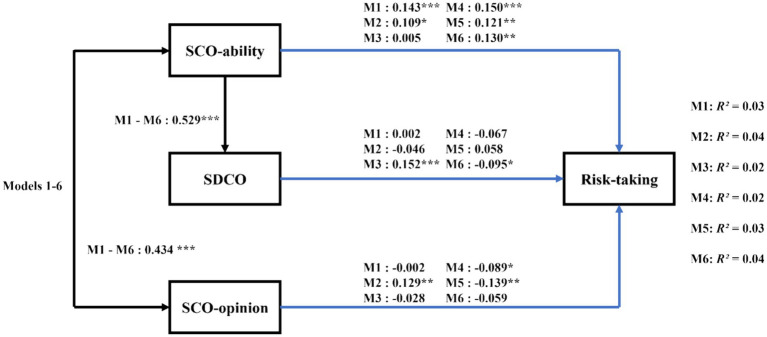
Diagram of Study 1: standardized path analysis for self-developmental competitive orientation. Black pathways illustrate findings consistent across all risk-taking categories; blue pathways depict findings specific to each risk category: M1 for total risks, M2 for social investment risks, M3 for recreational risks, M4 for ethical risks, M5 for gambling risks, M6 for health risks. R^2^ indicates the variance proportion in each risk type explained by the independent variables in each respective model. Sex was included in the study but not displayed in this figure for clarity. Solid lines represent significant paths (*p < 0.05, **p < 0.01, ***p < 0.001). SCO, social comparison orientation; SDCO, self-developmental competitive orientation.

Table 2 displays the bivariate correlations between the studied variables. Our analysis involved path analysis with observed variables across six proposed models. Each model demonstrated adequate compatibility with the data, and there was a high degree of similarity in fit among these models (e.g., overall risk-taking: χ^2^(2) = 2.83, *p* = 0.06, CFI = 0.98, SRMR = 0.03, RMSEA = 0.05). Overall, across all models, there was a strong correlation between SCO-ability and SCO-opinion. SCO-ability was a significant predictor of the HCO variable, and collectively, SCO-ability, SCO-opinion, and HCO were predictors of the risk-taking variable (Figure 2). Specifically, the results revealed that SCO-ability positively predicts SDCO (*B* = 0.529, *SE* = 0.085, *t* = 6.211, *p* = 0.000, 95%CI = [0.362, 0.701]). SCO-ability positively predicted overall risk-taking (*B* = 0.143, *SE* = 0.042, *t* = 3.394, *p* = 0.001, 95%CI = [0.060, 0.223]), as well as risk-taking in the social investment (*B* = 0.109, *SE* = 0.045, *t* = 2.419, *p* = 0.016, 95%CI = [0.022, 0.199]), ethical (*B* = 0.150, *SE* = 0.040, *t* = 3.714, *p* = 0.000, 95%CI = [0.070, 0.228]), gambling (*B* = 0.121, *SE* = 0.040, *t* = 3.043, *p* = 0.002, 95%CI = [0.042, 0.197]), and health/safety (*B* = 0.130, *SE* = 0.044, *t* = 2.978, *p* = 0.003, 95%CI = [0.044, 0.214]) domains (Figure 2). SCO-opinion positively predicted risk-taking in the social investment domain (*B* = 0.129, *SE* = 0.048, *t* = 2.703, *p* = 0.007, 95%CI = [0.041, 0.227]), but negatively predicted risk-taking in the ethical (*B* = −0.089, *SE* = 0.043, *t* = −2.099, *p* = 0.036, 95%CI = [−0.172, −0.007]) and gambling (*B* = −0.139, *SE* = 0.044, *t* = −3.164, *p* = 0.002, 95%CI = [−0.227, −0.054]) domains. SDCO positively predicted risk-taking in the recreational domain (*B* = 0.152, *SE* = 0.044, *t* = 3.449 *p* = 0.001, 95%CI = [0.065, 0.236]), but negatively predicted risk-taking in the health/safety domain (*B* = −0.095, *SE* = 0.043, *t* = −2.222, *p* = 0.026, 95%CI = [−0.179, −0.011]) (Figure 2).

**Table 2:** Study 1: means, standard deviations, Cronbach’s alpha coefficients, and the bivariate relations among the variables from the self-developmental competitive orientation model on student sample (*N* = 622).

	1	2	3	4	5	6	7	8	9
SCOA									
SCOO	0.396***								
SDCO	0.209***	0.261***							
(R_all)	0.139**	0.062	0.064						
(R_socialinvestment)	0.147**	0.166***	0.038	0.771***					
(R_recreational)	0.026	0.016	0.153***	0.751***	0.412***				
(R_ethical)	0.099*	−0.046	−0.051	0.599***	0.197***	0.347***			
(R_gambling)	0.076	−0.072	0.066	0.405***	−0.019	0.304***	0.384***		
(R_health)	0.083*	−0.022	−0.036	0.619***	0.331***	0.298***	0.387***	0.178***	
Mean (*SD*)	3.37(0.72)	3.88(0.67)	3.68(1.06)	2.56(0.49)	3.32(0.73)	2.34(0.72)	1.75(0.63)	1.78(0.80)	2.70(0.78)
Cronbach’s Alpha	0.74	0.64	0.85	0.86	0.85	0.70	0.71	0.75	0.55

SCOA, Social comparison orientation – ability; SCOO, Social comparison orientation – opinion; SDCO, self-developmental competitive orientation; R, Risk-taking; *p < 0.05, **p < 0.01, ***p < 0.001.

Additionally, our advanced bias-corrected bootstrap method, applied to evaluate the indirect effects of SCO-ability on various propensity for risk-taking via SDCO, revealed significant indirect impacts. Specifically, the effect of SCO-ability on overall risk-taking was found to be significant (*B* = 0.025, *SE* = 0.015, 95%CI = [0.004, 0.063]), as well as its effects on recreational risk-taking (*B* = 0.080, *SE* = 0.027, 95%CI = [0.034, 0.143]) and health/safety risk-taking (*B* = −0.050, *SE* = 0.025, 95%CI = [−0.106, −0.008]) through HCO.

## 3 Study 2

Study 2 aimed to explore how generalizable the findings obtained from the university student sample were when applied to a sample of adult workers.

### 3.1 Method

#### 3.1.1 Participants and procedure

The participant group in Study 2 consisted of 717 adult workers, with 368 males, recruited through WJX. Their ages varied from 18 to 50 years, with an average age of 27.92 and a standard deviation of 5.11. The methodologies employed were identical to those used in Study 1.

#### 3.1.2 Measures (see Tables 3, 4 for descriptive statistics and Cronbach’s alphas)

All assessments in Study 2 were conducted identically to those in Study 1.

### 3.2 Data analytic strategies

Our data analyses were the same as those in Study 1.

### 3.3 Results

#### 3.3.1 Relation among SCO, HCO, and risk-taking

**Figure 3:**
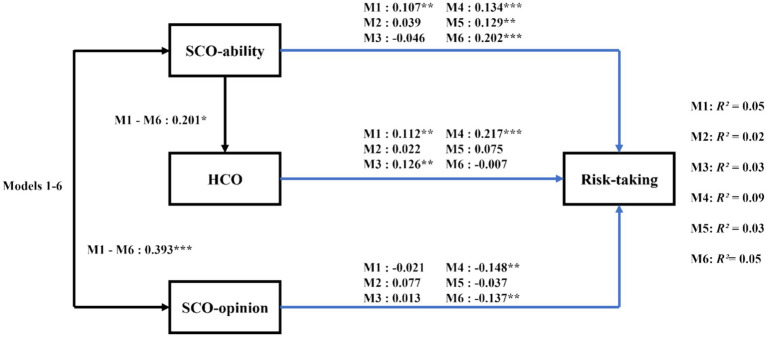
Diagram of Study 2: standardized path analysis for hypercompetitive orientation. Black pathways illustrate findings consistent across all risk-taking categories; blue pathways depict findings specific to each risk category: M1 for total risks, M2 for social investment risks, M3 for recreational risks, M4 for ethical risks, M5 for gambling risks, M6 for health risks. R^2^ indicates the variance proportion in each risk type explained by the independent variables in each respective model. Sex was included in the study but not displayed in this figure for clarity. Solid lines represent significant paths (*p < 0.05, **p < 0.01, ***p < 0.001). SCO, social comparison orientation; HCO, hypercompetitive orientation.

Table 3 presents the bivariate correlations among the variables. In this section, we analyzed six hypothesized models using path analysis with observed variables. Each model demonstrated acceptable fit to the data, with a high degree of similarity in fit across all six models (e.g., overall risk-taking: χ^2^(2) = 0.48, *p* = 0.62, CFI = 1.00, SRMR = 0.01, RMSEA = 0.00). In each of the models, SCO-ability and SCO-opinion were highly correlated, SCO-ability predicted the HCO variable, and SCO-ability, SCO-opinion, and HCO predicted a risking-taking variable (Figure 3). Specifically, the results indicated that SCO-ability positively predicts HCO (*B* = 0.201, *SE* = 0.090, *t* = 2.227, *p* = 0.026, 95%CI = [0.021, 0.375]). SCO-ability positively predicted overall risk-taking (*B* = 0.107, *SE* = 0.040, *t* = 2.658, *p* = 0.008, 95%CI = [0.027, 0.186]), as well as risk-taking in the ethical (*B* = 0.134, *SE* = 0.037, *t* = 3.614, *p* = 0.000, 95%CI = [0.062, 0.207]), gambling (*B* = 0.129, *SE* = 0.040, *t* = 3.252, *p* = 0.001, 95%CI = [0.050, 0.205]) and health/safety (*B* = 0.202, *SE* = 0.044, *t* = 4.557, *p* = 0.000, 95%CI = [0.113, 0.288]) domains (Figure 3). SCO-opinion negatively predicted risk-taking in the ethical (*B* = −0.148, *SE* = 0.046, *t* = −3.229, *p* = 0.001, 95%CI = [−0.237, −0.058]) and health/safety (*B* = −0.137, *SE* = 0.043, *t* = −3.230, *p* = 0.001, 95%CI = [−0.222, −0.053]) domains. HCO positively predicted overall risk-taking (*B* = 0.112, *SE* = 0.042, *t* = 2.647, *p* = 0.008, 95%CI = [0.031, 0.197]), as well as risk-taking in the recreational (*B* = 0.126, *SE* = 0.042, *t* = 2.985, *p* = 0.003, 95%CI = [0.042, 0.208]) and ethical (*B* = 0.217, *SE* = 0.041, *t* = 5.345, *p* = 0.000, 95%CI = [0.138, 0.297]) domains (Figure 3).

**Table 3:** Study 2: Means, standard deviations, Cronbach’s alpha coefficients, and the bivariate relations among the variables from the hypercompetitive orientation model on work sample (*N* = 717).

	1	2	3	4	5	6	7	8	9
SCOA									
SCOO	0.389***								
HCO	0.340***	0.081*							
(R_all)	0.140***	0.033	0.158***						
(R_socialinvestment)	0.078*	0.096*	0.047	0.783***					
(R_recreational)	0.005	0.010	0.122**	0.732***	0.397***				
(R_ethical)	0.152***	−0.076*	0.257***	0.502***	0.090*	0.313***			
(R_gambling)	0.141***	0.020	0.118**	0.366***	−0.013	0.215***	0.300***		
(R_health)	0.150***	−0.055	0.062	0.628***	0.318***	0.343***	0.350***	0.186***	
Mean (SD)	3.28(0.85)	3.93(0.68)	2.54(0.95)	2.51(0.46)	3.23(0.77)	2.34(0.65)	1.67(0.57)	1.91(0.81)	2.54(0.74)
Cronbach’s Alpha	0.86	0.64	0.73	0.84	0.86	0.62	0.68	0.70	0.52

SCOA, Social comparison orientation – ability; SCOO, Social comparison orientation – opinion; HCO, hypercompetitive orientation; SDCO, self-developmental competitive orientation; R, Risk-taking; *p < 0.05, **p < 0.01, ***p < 0.001.

Moreover, our further analysis using a bias-corrected bootstrap method to test the indirect effects of SCO-ability on risk-taking via HCO revealed significant indirect effects of SCO-ability on different types of risk-taking. Specifically, the effects on overall risk-taking were significant (*B* = 0.023, *SE* = 0.014, 95%CI = [0.003, 0.060]), as were those on recreational risk-taking (*B* = 0.025, *SE* = 0.014, 95%CI = [0.004, 0.064]) and ethical risk-taking (*B* = 0.044, *SE* = 0.022, 95%CI = [0.007, 0.094]) through the influence of HCO.

#### 3.3.2 Relation among SCO, SDCO, and risk-taking

**Figure 4:**
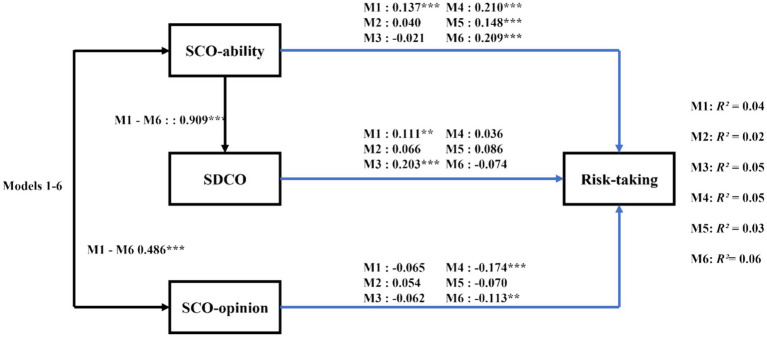
Diagram of Study 2: standardized path analysis for self-developmental competitive orientation. Black pathways illustrate findings consistent across all risk-taking categories; blue pathways depict findings specific to each risk category: M1 for total risks, M2 for social investment risks, M3 for recreational risks, M4 for ethical risks, M5 for gambling risks, M6 for health risks. R^2^ indicates the variance proportion in each risk type explained by the independent variables in each respective model. Sex was included in the study but not displayed in this figure for clarity. Solid lines represent significant paths (*p < 0.05, **p < 0.01, ***p < 0.001). SCO, social comparison orientation; SDCO, self-developmental competitive orientation.

Table 4 presents the bivariate correlations among the variables. In this section, we analyzed six hypothesized models using path analysis with observed variables. Each model demonstrated acceptable fit to the data, with a high degree of similarity in fit across all six models (e.g., overall risk-taking: χ^2^(2) = 3.76, *p* = 0.02, CFI = 0.98, SRMR = 0.03, RMSEA = 0.06). Overall, in each of the models, SCO-ability and SCO-opinion were highly correlated, SCO-ability predicted the SDCO variable, and SCO-ability, SCO-opinion, and SDCO predicted risk-taking variables (Figure 4). Specifically, the results revealed that SCO-ability positively predicts SDCO (*B* = 0.909, *SE* = 0.113, *t* = 8.055, *p* = 0.000, 95%CI = [0.708, 1.154]). SCO-ability positively predicted overall risk-taking (*B* = 0.137, *SE* = 0.038, *t* = 3.661, *p* = 0.000, 95%CI = [0.063, 0.210]), as well as risk-taking in the ethical (*B* = 0.210, *SE* = 0.035, *t* = 5.985, *p* = 0.000, 95%CI = [0.140, 0.278]), gambling (*B* = 0.148, *SE* = 0.037, *t* = 3.982, *p* = 0.000, 95%CI = [0.075, 0.220]), and health/safety (*B* = 0.209, *SE* = 0.042, *t* = 5.021, *p* = 0.000, 95%CI = [0.125, 0.289]) domains (Figure 4). SCO-opinion negatively predicted risk-taking in the ethical (*B* = −0.174, *SE* = 0.048, *t* = −3.629, *p* = 0.000, 95%CI = [−0.267, −0.081]) and health/safety (*B* = −0.113, *SE* = 0.043, *t* = −2.597, *p* = 0.009, 95%CI = [−0.198, −0.026]) domains. SDCO positively predicted risk-taking in the overall risk-taking domain (*B* = 0.111, *SE* = 0.040, *t* = 2.796, *p* = 0.005, 95%CI = [0.034, 0.189]), as well as risk-taking in the recreational domain (*B* = 0.203, *SE* = 0.039, *t* = 5.194, *p* = 0.000, 95%CI = [0.124, 0.278]) (Figure 4).

**Table 4:** Study 2: Means, standard deviations, Cronbach’s alpha coefficients, and the bivariate relations among the variables from the self-developmental competitive orientation model on work sample (*N* = 717).

	1	2	3	4	5	6	7	8	9
SCOA									
SCOO	0.389***								
SDCO	0.235***	0.375***							
(R_all)	0.140***	0.033	0.134***						
(R_socialinvestment)	0.078*	0.096*	0.103**	0.783***					
(R_recreational)	0.005	0.010	0.189***	0.732***	0.397***				
(R_ethical)	0.152***	−0.076*	0.031	0.502***	0.090*	0.313***			
(R_gambling)	0.141***	0.020	0.097**	0.366***	−0.013	0.215***	0.300***		
(R_health)	0.150***	−0.055	−0.049	0.628***	0.318***	0.343***	0.350***	0.186***	
Mean (SD)	3.28(0.85)	3.93(0.68)	3.85(1.12)	2.51(0.46)	3.23(0.77)	2.34(0.65)	1.67(0.57)	1.91(0.81)	2.54(0.74)
Cronbach’s Alpha	0.86	0.64	0.84	0.84	0.86	0.62	0.68	0.70	0.52

SCOA, Social comparison orientation – ability; SCOO, Social comparison orientation – opinion; SDCO, self-developmental competitive orientation; R, Risk-taking; *p < 0.05, **p < 0.01, ***p < 0.001.

Moreover, our subsequent bias-corrected bootstrap method revealed that the indirect effects of SCO-ability on overall risk-taking (*B* = 0.101, *SE* = 0.038, 95%CI = [0.032, 0.182]) and recreational risk-taking (*B* = 0.185, *SE* = 0.042, 95%CI = [0.112, 0.278]) via SDCO were significant.

## 4 Discussion

The SCO-ability and TC constitute deep conceptual overlap and both of them have been shown to have important influences on psychological functions, such as risk-taking (Buunk et al., 2007; Keresztes et al., 2015). Here we find that SCO-ability is positively correlated with TC, but also that SCO-ability is the antecedent of TC, consistent with recent theory (Garcia et al., 2013, 2020; Tor and Garcia, 2023). However, evidence on the link between SCO-ability and TC, as well as their utility in predicting both domain-general and domain-specific risk-taking, remains limited. Given that TC has been argued to be a multi-dimensional trait (Ryckman et al., 1990, 1996; Houston et al., 2002; Newby and Klein, 2014; Orosz et al., 2018), the present study expanded on prior work by examining to what extent SCO-ability might be linked with two differentiated measures of TC (HCO and SDCO) and whether HCO and SDCO would differentially mediate the relation between SCO-ability and overall and domain-specific risk-taking. Below, we will discuss the possible implications of our findings for deepening the understanding of the link between SCO, TC, and risk-taking.

Regarding the link between SCO-ability and the two facets of TC, we found that SCO-ability and HCO were positively correlated. Path models further revealed that SCO-ability predicted HCO. HCO refers to a strong tendency or desire by individuals to compete and win as a means to maintain or enhance feelings of self-worth. Our finding that SCO-ability predicts HCO suggests that the tendency toward manipulating and exploiting others as a comparative standard, facilitates the desire to have a better performance than others, in order to demonstrate one’s superiority. In other words, individuals with high SCO-ability were more concerned with dominating or with outcomes of being superior to others (Garcia et al., 2013, 2020; Orosz et al., 2018). Similarly, we found that SCO-ability also predicted SDCO. SDCO refers to the desire to compete with others for personal development, but the primary focus is not the outcome (i.e., winning), but rather the enjoyment and mastery of the task. Our observation that SCO-ability predicts SDCO implies that a tendency toward using others as a comparative standard facilitates the desire to gauge one’s abilities or motivate oneself to succeed. These findings are consistent with the proposal of Festinger (1954) and the recent theorizing by Garcia et al. (2013, 2020). As documented by the social comparison theory of Festinger (1954), people naturally rely on social comparison when processing information about themselves and others, and are propelled by a basic “unidirectional upward drive” (Garcia et al., 2013). In order to minimize the gaps between themselves and others, individuals are more likely to demonstrate higher competitiveness, either because of too great a concern with respect to the result of competition, or because of competitiveness for self-development purposes (Griffin-Pierson, 1990; Ryckman et al., 1994, 1996, 1997; Mudrack et al., 2012). In this sense, these results extend prior work (Liu et al., 2021) and take a further step toward understanding the link between SCO and different aspects of TC.

Meanwhile, alongside recent research (Liu et al., 2021), the overall results for SCO and risk-taking illustrated stronger relations for SCO-ability than SCO-opinion. Regarding the link between SCO-ability, HCO, and risk-taking, we found that SCO-ability positively correlated with overall risk-taking and the gambling and health/safety risk-taking in both studies (and the social investment risk-taking in Study 1 alone and the ethical risk-taking in Study 2 alone). In contrast, SCO-opinion did not consistently correlate with any of the domain-specific risk-taking indicators across both studies (but only showed a positive correlation with risk-taking in the social investment domain only in Study 1). HCO showed positive correlations with overall risk-taking and risk-taking in the recreational and ethical domains in both studies (and in the gambling domain in Study 1 alone). Mediation analyses revealed that HCO played a mediating role in the link between SCO-ability and both overall risk-taking and recreational and ethical risk-taking in both studies (and gambling risk-taking for Study 1 alone). This result was consistent between both college students and adult workers. This indicates that the mediating effect of HCO on the predictive utility of SCO-ability in risk-taking is stable across young and middle-aged people, at least concerning the risk transmission that results from social comparison, and may be promoted by excessive competition, which may ultimately lead to gambling, immorality, and hedonic behaviors (Hangen et al., 2016; Filippin and Gioia, 2018; Gärling et al., 2019). Regarding the link between SCO-ability, SDCO, and risk-taking, SCO-ability positively correlated with overall risk-taking and in the ethical, gambling, and health/safety risk-taking in both studies (and in the social investment domain in Study 1 alone). SDCO showed positive correlations with risk-taking in the recreational domain in both studies (and in the overall risk-taking domain in Study 2 alone). Our mediation analyses found that SDCO played a mediating role in the link between SCO-ability and risk-taking in the recreational domain in both studies (and overall risk-taking for Study 2 alone). Risk-taking in the recreational domain is driven by intrinsic motivation, and consequently has been shown to be chosen for promoting personal growth and well-being (Gracia-Garrido et al., 2022; Hernández-Méndez et al., 2024). Our finding that SCO-ability only predicted recreational risk-taking through SDCO in both studies is not surprising. Specifically, social comparison not only involves a straightforward cognitive assessment but also produces self-oriented comparisons, which motivate the individuals’ desire for excellence and favor them engaging in competition (Griffin-Pierson, 1990; Ryckman et al., 1997; Kayhan, 2003; Gärling et al., 2021). Therefore, individuals become more vulnerable to risk-taking because taking risks is frequently considered to be a tempting strategy to improve one’s chances of achieving success (Hangen et al., 2016; Filippin and Gioia, 2018). Hence, an enduring motivation to do or be better than the other (TC), favors risk-taking.

The findings above advance our understanding of the relation between competition and risk-taking by using differentiated measures of TC (HCO and SDCO). Moreover, our findings suggest that HCO is more strongly related to risk-taking than SDCO, which supports the claim in previous research (Harris et al., 2015; Liu et al., 2021). In summary, this study contributes to a growing body of literature emphasizing the existence of a strong nexus of interactions among social comparison, TC, and risk-taking (Zhu et al., 2016; Filippin and Gioia, 2018; Gärling et al., 2021; Liu et al., 2021; Li et al., 2023; Schwerter, 2023).

### 4.1 Potential limitation

There were several potential limitations to this study that need to be pointed out. First, since both HCO and SDCO were partial, rather than full mediators, of the link between SCO-ability and risk-taking across domains, this suggests that other facets of TC such as “competition avoidance” may act as potential mediator variables (Ryckman et al., 2009). “Competition avoidance” represents a neurotic aspect of social competitive orientation marked by individuals’ fear of both success and failure in competition, which leads them to avoid competitive situations. It should be interesting to incorporate this additional dimension of TC in future studies, which may enrich our understanding of the mediating role of TC in the relation between SCO and risk-taking. Second, although we adopted a two-time point design, the time intervals between data collection were relatively short. Therefore, additional studies adopting a longitudinal design are needed to examine the association between SCO-ability, multidimensional competitiveness, and risk-taking over longer time periods. Third, although we tapped into the underlying process through which SCO-ability predicted TC and thus predicted risk-taking, we merely focused on the implications of individuals’ propensity for risk-taking. There are contextual factors that require continued empirical inquiry. For instance, aggression-hostility, a personality trait linked with hypercompetitiveness, has been found to predict gambling behaviors across different contexts, indicating a strong association with risk-taking (Thornton et al., 2011). This relationship is further complicated by situational factors such as real versus hypothetical rewards, where real monetary incentives increase loss aversion and thus alter risk preferences (Xu et al., 2016). Time pressure in competitive settings like auctions has been shown to increase financial risks (Adam et al., 2015). Moreover, the presence of an audience can either increase competitive pressure or provide a social shield, leading to more conservative decisions (Garcia et al., 2013; Lemoine and Roland-Lévy, 2017). Researchers also need to reflect on the classic distinction between risk and uncertainty as defined by Knight (1921), where risk involves known probabilities, and uncertainty is marked by unknown outcomes and their probabilities, impacting decision-making significantly. These dynamics underscore the complex interplay between individual psychological traits and external environmental factors in shaping risk-taking behaviors, necessitating a multidimensional approach to understanding these behaviors. To this end, future research may take the effect of situation-based competition on risk-taking across domains into account. Fourth, our present study only involved Chinese participants in both studies. Thus, it remains unclear if the findings can be generalized to other countries. While acknowledging the limitations in generalizability due to the exclusive inclusion of Chinese participants, it is pertinent to consider the potential variability in results across different cultural contexts. Research suggests that cultural dimensions such as uncertainty avoidance significantly influence individual behaviors (Lonner et al., 1980). In cultures characterized by high uncertainty avoidance, individuals tend to be more risk-averse and less inclined to take risks in pursuit of potential rewards (Hofstede, 2001). Therefore, it would be beneficial to explore how these cultural factors might moderate the observed associations between social comparison orientation (SCO) or temporal comparison (TC) and risk-taking behaviors. Discussing these cultural considerations could enhance our understanding of the findings’ universality and applicability, suggesting valuable directions for further research across diverse cultural settings. Last but not least, it is imperative to acknowledge a pertinent observation regarding our methodology of determining our sample size. While our methodology is deemed acceptable and justified, it is noteworthy that power analysis, as an alternative approach for determining sample size, warrants recognition. The power analysis as an alternative and widely employed method of determining sample sizes should be taken into account in future studies. Despite these limitations, we believe that our findings further open up the possibility of gaining further insights into how SCO is linked to TC and how these two variables predict risk-taking.

## 5 Conclusion

Our aim with the current study was to contribute to an understanding of how SCO-ability predicts different facets of TC and the utility of these two variables in predicting risk-taking across domains in different age groups. We found that SCO-ability predicts two differentiated measures of TC, consistent with the proposal of Festinger (1954) and recent theorizing (Garcia et al., 2013, 2020). Meanwhile, the current research reveals that SCO-ability predicts specific types of risk-taking among both college students and adult workers. Furthermore, HCO is more strongly related to risk-taking than SDCO, and HCO and SDCO differentially mediated the relation between SCO-ability and risk-taking. This study may have substantial significance for deepening our understanding of the relation between SCO-ability and competition, and domain-specific risk-taking.

## Data availability statement

The data and materials for the present experiment are available upon request to the corresponding author.

## Ethics statement

The studies involving humans were approved by the Ethical Review Board of the University of Nanjing. The studies were conducted in accordance with the local legislation and institutional requirements. The participants provided their written informed consent to participate in this study.

## Author contributions

YW: Formal analysis, Investigation, Methodology, Software, Visualization, Writing – original draft. AE: Methodology, Supervision, Writing – review & editing. ED: Writing – review & editing. YL: Conceptualization, Data curation, Funding acquisition, Investigation, Methodology, Project administration, Resources, Writing – review & editing.

## Footnotes

^1^ https://www.statmodel.com
